# Female and male mouse lung group 2 innate lymphoid cells differ in gene expression profiles and cytokine production

**DOI:** 10.1371/journal.pone.0214286

**Published:** 2019-03-26

**Authors:** Laura Mathä, Hanjoo Shim, Catherine A. Steer, Yi Han Yin, Itziar Martinez-Gonzalez, Fumio Takei

**Affiliations:** 1 Terry Fox Laboratory, British Columbia Cancer, Vancouver, British Columbia, Canada; 2 Interdisciplinary Oncology Program, Faculty of Medicine, University of British Columbia, Vancouver, British Columbia, Canada; 3 Department of Pathology and Laboratory Medicine, University of British Columbia, Vancouver, British Columbia, Canada; Albany Medical College, UNITED STATES

## Abstract

Epidemiological studies have shown sex differences in prevalence of non-allergic asthma. Recent reports demonstrated negative effects of androgen signaling on group 2 innate lymphoid cells (ILC2s), explaining a potential mechanism behind sex bias in asthma prevalence. To further understand sex-related differences in ILC2 functions and ILC2 intrinsic or lung environmental mechanisms behind it, we have investigated the effects of sex and age on lung ILC2 function, the amounts of ILC2-activating cytokines in the lung and gene expression profiles of male and female ILC2s. Flow cytometric analyses of naive male and female mouse lung ILC2s showed no difference in their numbers. However, upon three daily intranasal IL-33 injections, lung ILC2s in postpubertal female mice expanded to a greater degree than male counterpart. In line with in vivo results, purified female mouse lung ILC2s produced more cytokines than male ILC2s upon in vitro stimulation. Gene expression profiles of purified naïve male and female ILC2s differed in 4% of the genes, and gene set enrichment analysis showed that female ILC2s are enriched for gene signatures of memory T cells. We did not observe similar degree of differences between female and male ILC2s after IL-33 stimulation. ILC2-activating cytokines including IL-33, IL-7 and TSLP were more highly expressed in whole lung homogenate samples prepared from naïve post pubertal female mouse lung than male mouse lung. Moreover, the differences in responsiveness of male and female ILC2s to IL-33 were not affected in IL-33-deficient mice. These results suggest that female ILC2s are more readily activated than male ILC2s due to their gene expression at the naïve state, which is potentially influenced by the lung environment.

## Introduction

We and others have previously identified Group 2 innate lymphoid cells (ILC2s) in mouse lungs [1, 2]. ILC2s are antigen non-specific innate lymphocytes that reside in mucosal tissues such as the lung. They are activated by epithelium derived cytokines including interleukin (IL) -33, IL-25, IL-7 and thymic stromal lymphopoietin (TSLP) and produce type 2 cytokines IL-5 and IL-13 [3]. IL-5 induces eosinophil infiltration [4], while IL-13 stimulates mucus hyperproduction [5], resulting in type 2 inflammation in the airways. They have been found to be elevated in asthmatic patients’ sputum [6], blood [7] and bronchoalveolar lavage fluid (BALF) [8].

Asthma is caused by chronic inflammation of the airways. It is estimated that approximately 300 million people suffer from asthma worldwide, but currently there is no cure. Epidemiological studies have shown a higher prevalence of non-allergic asthma in women than men [9]. Multiple studies have also described more pronounced type 2 responses in females compared to males in OVA-induced mouse models of asthma [10–12]. Recent studies have shown sex-related differences in mouse ILC2 development and responsiveness. Warren et al. reported that ILC2s isolated from female mouse lungs were more responsive upon ex vivo cytokine stimulation compared to male ILC2s [13]. Laffont et al. reported that female mice have higher numbers of ILC2s and ILC2 progenitors due to inhibitory effects of androgen signaling on ILC2 development [14]. Cephus et al. also found that adult female mice have higher numbers of ILC2s in the lung than male mice and female ILC2s proliferate more than male ILC2s in response to IL-2 [15]. Interestingly, Kadel et al. demonstrated that there is a killer-cell lectin like receptor G1 (KLRG1) negative population of ILC2s in females that increases with age and partly contributes to the higher number of ILC2s in female lungs compared to male lungs in which this population is largely absent. They also showed that androgen signaling inhibits differentiation of bone marrow ILC precursors into ILC2s [16]. In contrast, Bartemes and colleagues reported a role of estrogen in the regulation of uterine but not lung ILC2s, demonstrating tissue-dependent effects of sex hormones on ILC2s [17].

To further understand the sex differences in ILC2 functions, we have investigated gene expression profiles of ILC2 and ILC2-activating cytokines in the lung of male and female mice. Here, we show that there is no significant difference in the numbers of ILC2s between naïve male and female mouse lungs, contradicting previous reports [14–16]. Post pubertal female lung ILC2s produce more cytokines upon activation than male counterparts. Gene expression analyses suggest that female ILC2s are enriched for gene signatures of memory T cells whereas pathway analysis also suggest that naïve female lung ILC2s are more metabolically active than male ILC2s. These results suggest that female lung ILC2s are more prone to be activated by IL-33 than male ILC2s due to intrinsic differences in gene expression.

## Materials and methods

### Mice

C57BL/6J (B6) and B6.*Il33*^-/-^ (obtained from KOMP) mice were maintained in the British Columbia Cancer Research Centre pathogen-free animal facility. B6.*Rag1*^-/-^ mice were purchased from the Jackson Laboratory. All animal use was approved by the animal care committee of the University of British Columbia and were maintained and euthanized in accordance with the guidelines of the Canadian Council on Animal Care. Briefly, mice were housed in static cages (4 mice maximum per cage) with cotton or crinkle paper nesting materials and a hiding place. They were fed low fat diet and water was provided per cage. To minimize animal suffering and distress, we anesthetized mice by isoflurane inhalation during intranasal injections and monitored them until they were fully recovered from anesthesia in a separate cage with heating mat underneath it. The mice were monitored daily during intranasal injections and one day after the last injections and their health status was assessed by their behaviour, appearance, hydration status, respiration, and presence/absence of any obvious pain. Their health and well-being were monitored daily by facility staff based on their appearance and behaviour. At the time of harvest, we anesthetized mice by isoflurane inhalation until they were unconscious and performed euthanasia by carbon dioxide asphyxiation. Mice were treated at the ages indicated in the text.

### Antibodies, reagents and flow cytometers

Fluorescein isothiocyanate (FITC)-conjugated anti-Ki67 (SolA15), Rag IgG2a kappa isotype control (eBR2a), PerCP-Cy5.5-conjugated anti-CD19 (1D3), NK1.1 (PK136), CD3ε (145-2C11), CD25 (PC61.5), Allophycocyanin (APC)-conjugated anti-FcεR1α (MAR-1), CCR9 (CW-1.2), Mouse IgG2a kappa isotype control (eBM2a), Alexa Fluor 700-conjugated anti-CD45.2 (104), CD11c (N418), eFluor 450-conjugated anti-CD3ε (145-2C11), CD4 (RM4-5), CD19 (1D3), CD11b (M1/70), TCRγδ (GL3), CD11c (N418), NK1.1 (PK136), TCRβ (H57-597), Gr1 (RB6-8C5), Ter119 (TER-119), Phycoerythrin (PE)-conjugated anti-CD127 (A7R34) and anti-GATA3 (TWAJ), PE-Cyanine7 conjugated anti-CD127 (A7R34) were purchased from Thermo Fisher Scientific (Waltham, MA). FITC-conjugated anti-ST2 (DJ8) was purchased from MD Bioproducts (Oakdale, MN), and FITC-conjugated anti-7/4 (7/4) was purchased from Abcam (Cambridge, United Kingdom).

V500 anti-CD45 (30-F11), BV605-conjugated anti-CD90.2 (53–2.1), and PE-conjugated Siglec-F (E50-2440) were purchased from BD Biosciences (Franklin Lakes, NJ). eFluor 780 (Thermo Fisher Scientific) was used to exclude dead cells. Recombinant IL-33 and TSLP were purchased from Thermo Fisher Scientific. ILC2s were identified as Lineage (CD3ε, CD4, CD19, CD11b, CD11c, TCRβ, TCRγδ, NK1.1, Gr1, Ter119)^-^CD45^+^Thy1(CD90)^+^CD127^+^ST2^+^ cells and eosinophils were identified as Lym (CD3ε, CD19, NK1.1)^-^7/4^-^SiglecF^+^CD11c^-^ cells. GATA3 and Ki67 expression was analyzed by a Foxp3 transcription factor staining buffer set kit (Thermo Fisher Scientific) according to manufacturer’s protocol. BD FACS Aria was used for cell sorting and BD Fortessa was used for flow cytometric analyses. Flowjo version 10 was used for data analyses.

### Primary leukocyte preparation

Single cell suspensions were prepared from the lungs as previously described [18]. They were counted using a hemocytometer, incubated in 2.4G2 mAb to block Fc receptors, and stained with flow cytometery antibodies to sort or analyze by fluorescence-activated cell sorting (FACS).

### ILC2 enrichment

Primary leukocyte cell suspension was enriched for ILC2s using Easy Sep Mouse ILC2 Enrichment Kit (STEMCELL Technologies) according to manufacturer’s protocol.

### In vivo stimulation

Mice were anesthetized by isoflurane inhalation and intranasal injections were given. Mice were given 3 daily intranasal administrations of 0.25 μg IL-33 in 40 μL PBS.

### In vitro stimulation

ILC2s were sorted from naïve female and male lungs after ILC2 enrichment and 1000 cells were cultured in 200 μL RPMI-1640 media containing 10% FBS, P/S, 50 mM 2ME, 5 ng/mL TSLP and IL-33. Culture supernatant was collected 48 hours later. For whole lung leukocytes cultures, single cell suspensions were prepared from B6 and *Rag1*^-/-^ mice and 500,000 cells were cultured as described above for 72 hours in presence of 10 ng/mL (B6) or 5 ng/mL (*Rag1*^-/-^) IL-33 and TSLP.

### Lung homogenate preparation and analyses

Lungs were collected from naive and IL-33 treated mice one day after three consecutive injections and homogenized in Hank’s Balanced Salt Solution (HBSS) with EDTA and Halt protease inhibitor cocktail (Thermo Fisher Scientific) at 200 mg/mL. After centrifugation at 800 xg for 20 min, supernatant was analyzed using IL-33, TSLP (Thermo Fisher Scientific) and IL-7 (Abcam) ELISA according to manufacturer’s protocols. Total protein was quantified using Protein quantification kit-rapid (Sigma Aldrich).

### Quantification of cytokines

BALF samples and in vitro culture supernatant were analyzed for IL-5, IL-13 and/or CCL3 using Thermo Fisher Scientific ELISA kits according to the manufacturer’s protocol.

### Immunohistochemistry

Immunohistochemical staining of lung sections were performed using a polyclonal goat anti-mouse IL-33 IgG (R&D systems, Minneapolis, MN) at 5 μg/mL (Vector Labs, Olean, NY) as described before [19].

### RNA extraction, microarray analyses and GSEA

Total RNA was extracted from FACS-purified ILC2s using TRIzol reagent (Thermo Fisher Scientific, Waltham, MA) according to manufacturer’s protocol. RNA quality check, cDNA preparation and microarray hybridization were performed at The Centre for Applied Genomics (Toronto, Canada). Briefly, RNA quality was assessed using Agilent 2100 Bioanalyzer and the samples with RNA integrity number (RIN) above 6 were selected for microarray analyses. RNA amplification and cDNA preparation was performed using GeneChip WT Pico Kit (Thermo Fisher Scientific). cDNA samples were hybridized to Thermo Fisher Scientific GeneChip Mouse Gene 2.0ST Array. Three samples per group were analyzed for gene expression profile by Flex Array 1.6.3 (Genome Quebec) after normalization using robust multi-array (RMA) algorithm. Gene set enrichment analysis was performed using GSEA software and the gene set collection C7 (immunological signatures) (http://software.broadinstitute.org/gsea/index.jsp).

### Visualization of cytoscape network analyses

Differentially expressed gene sets (p < 0.05, no fold difference cut-off was applied) were analyzed using Cytoscape (v3.6.1) plugin BiNGO (v.3.0.3) to identify over-represented “GO biological process” terms [20]. The hypergeometric test was used to measure the statistical significance of the enrichments and the Benjamini & Hochberg method (p < 0.05) was used to correct p-values. Resulting BiNGO output files were then visualized as functional overlapping networks using the Cytoscape plugin Enrichment Map (v.3.1.0) with the following parameters: P-value cut-off of 0.001, Q-value cut-off of 0.05, and Jaccard Coefficient cut-off of 0.25 [21]. The nodes comprising the network were then clustered using the plugin Autoannotate (v.1.2) and the clusters were labelled manually by revising labels generated by the plugin.

### Statistics

GraphPad Prism 7 was used for data analyses. Unpaired Student’s t test was used to determine statistical significance, with a P value < 0.05 being significant. Data in graphs represent the mean +/- SEM (*P<0.05, **P<0.01, ***P<0.005, ****P<0.0001, ns, not significantly different [P>0.05]).

## Results

### Age and sex dependent ILC2 responses to IL-33

**Fig 1:**
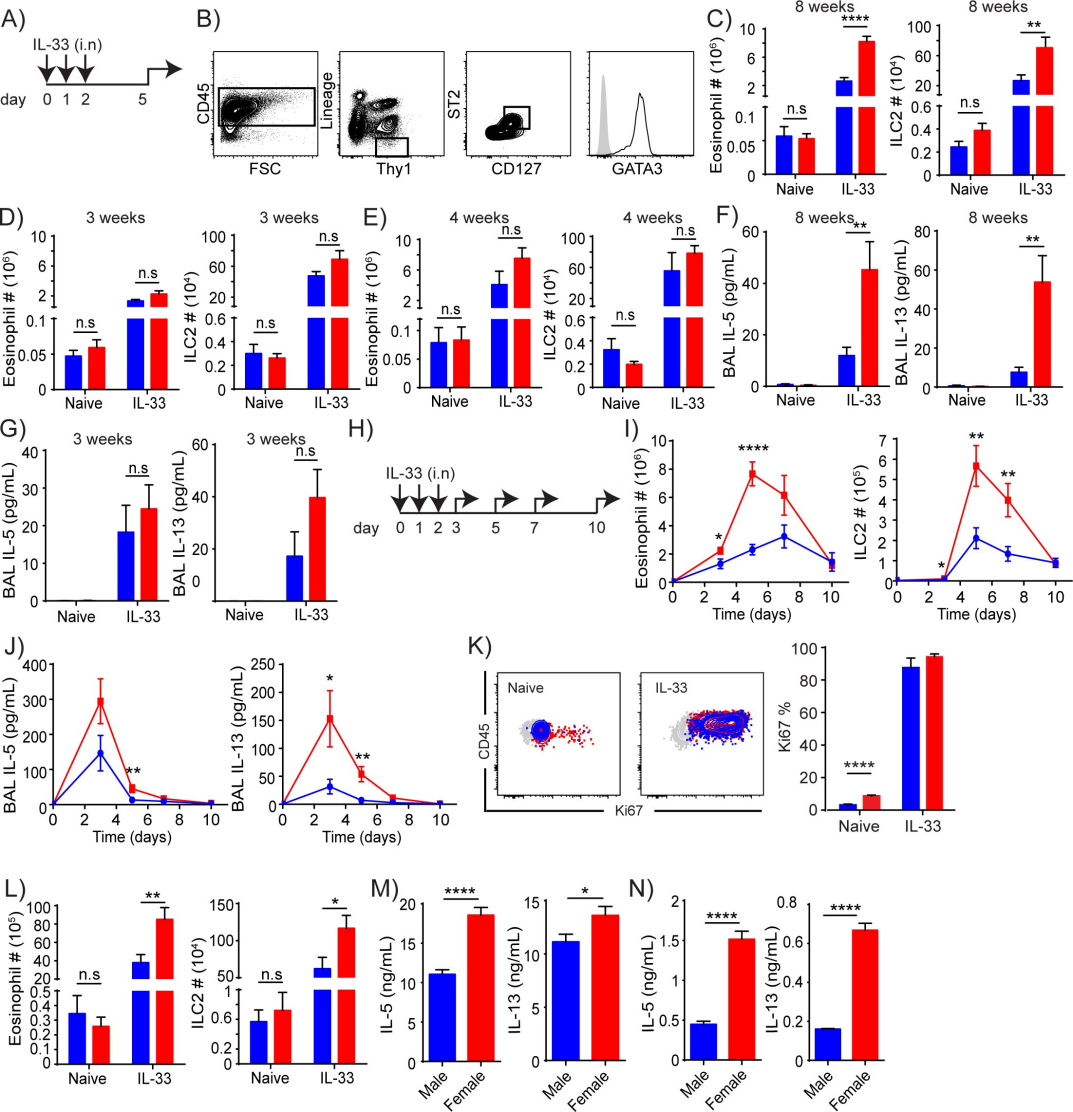
Female ILC2s respond more vigorously to IL-33 stimulation than male ILC2s. (A) Treatment scheme for in vivo experiments. (B) Gating strategy to identify ILC2s. They were identified as Lineage (CD3ε, CD4, CD19, CD11b, CD11c, TCRβ, TCRγδ, NK1.1, Gr1, Ter119)^-^CD45^+^Thy1(CD90)^+^CD127^+^ST2^+^ cells and confirmed for GATA3 expression. Grey = fluorescence-minus-one control, black line = ILC2. (C-E) Eosinophil and ILC2 numbers in untreated or IL-33 treated 8 week- (C), 3 week- (D) and 4 week- (E) old mice 3 days after 3 consecutive IL-33 injections. (F,G) BALF cytokines in untreated or IL-33 treated 8 week- (F) and 3 week- (G) old mice 3 days after 3 consecutive IL-33 injections. (H) Treatment scheme for in vivo time-course analyses. (I, J) Eosinophil and ILC2 numbers (I) and BALF cytokines (J) were quantified at day 0 (naive) and then 1 day (day 3), 3 days (day 5), 5 days (day 7) and 8 days (day 10) after 3 consecutive IL-33 injections. (K) Ki67 staining of male (blue) and female (red) ILC2s before and 1 day after IL-33 injections. Grey = Isotype control. (L) Eosinophil and ILC2 numbers in untreated or IL-33 treated 8 week-old *Rag1*^-/-^ mice 3 days after 3 consecutive IL-33 administrations. (M, N) Amounts of IL-5 and IL-13 in supernatant collected from male or female B6 (M) or *Rag1*^-/-^ (N) whole lung leukocytes cultures stimulated with 10 ng/ml (M) or 5 ng/ml (N) IL-33 and TSLP for 72 hours. Red = female, blue = male. Data represented are mean ± SEM of more than 3 (C, D, F, G, I, J) or 2 (E, K, L) experiments with 5–14 (C, D, F, G), 4–19 (I, J), 4–6 (E), 5–7 (K) or 3–7 (L) mice per group, or mean ± SEM of 7–10 (M) or 4–5 (N) replicates per group. Two-tailed Student’s t-test was used to determine statistical significance, with a P value <0.05 being significant. *P<0.05, **P<0.01, ****P<0.0001, ns, not significantly different [P>0.05].

We gave three daily intranasal injections of IL-33 (Fig 1A) into male and female mice of various ages and found greater numbers of ILC2s (2.7 fold, Gating strategy in Fig 1B) and eosinophils (3.3 fold) in female lungs compared to male lungs of 8 week-old mice (Fig 1C), but not 3 week- (Fig 1D) or 4 week-old mice (Fig 1E). In contrast, the numbers of ILC2s and eosinophils in naïve male and female lungs were not significantly different at any age (Fig 1C–1E), contradicting the recent report by Laffont et al., Cephus et al. and Kadel et al. We have also analyzed the percentages of various cell types in the lungs of these mice. Most cell types showed no difference between male and female in naive state. After IL-33 injections, eosinophils expanded more in female than male mice, which consisted approximately 40 and 50% of CD45^+^ cells in male and female lungs, respectively. ILC2 percentages were similar between male and female at all ages tested (S1A–S1C Fig). The amounts of IL-5 and IL-13 in BALF from the IL-33 treated 8 week-old female mice were also significantly higher compared to age-matched treated male mice (Fig 1F) but the difference was not seen in 3 week-old mice (Fig 1G). The time-course analyses (Fig 1H) of ILC2s and eosinophils in the lung as well as cytokines in BALF showed that female ILC2s expand and are activated to a greater degree by IL-33 than male ILC2s (Fig 1I and 1J). We also analyzed the expression of the proliferation marker Ki67 in naïve and IL-33 treated male and female ILC2s one day after three consecutive injections. Interestingly, significantly higher percentages of naïve female lung ILC2s expressed Ki67 compared to male lung ILC2s. However, once they are stimulated with IL-33, the difference was no longer present (Fig 1K). As ILC2s are thought to be regulated by regulatory T cells (Treg), which express the IL-33 receptor ST2 [22, 23], and differences between female and male Tregs have also been reported [24, 25], we tested *Rag1*^*-/-*^ mice, which are deficient in T and B cells. Intranasal injections of IL-33 into *Rag1*^*-/-*^ mice (8 weeks old) resulted in significant differences in the numbers of ILC2s and eosinophils in the lung between males and females, similar to B6 wild type mice (Fig 1L), indicating that the ILC2 difference between sex is independent of Tregs. These results were confirmed by in vitro IL-33 and TSLP stimulation of whole lung leukocytes from B6 (Fig 1M) and *Rag1*^-/-^ (Fig 1N) mice.

### ILC2-activating cytokines in the lung

**Fig 2:**
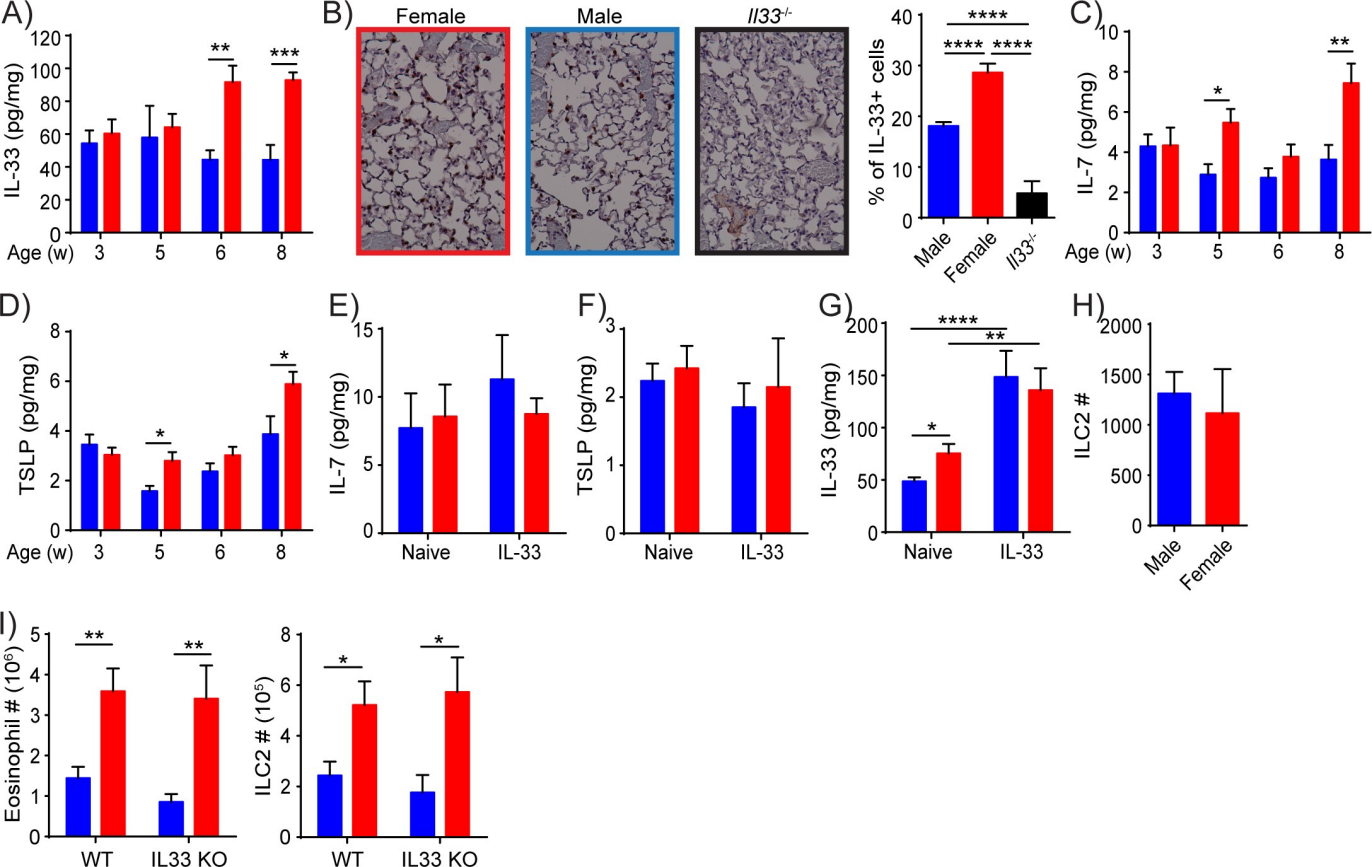
Epithelium derived cytokines are differentially expressed in female and male lungs. (A) The amounts of IL-33 in naïve mouse whole lung homogenates were measured using ELISA at different ages. (B) Immunohistochemical analysis and quantification of IL-33 in naïve 8 week-old lungs (200x magnification). (C, D) The amounts of IL-7 (C) and TSLP (D) in naïve mouse whole lung homogenates were measured as in A. (E-G) The amounts of IL-7 (E), TSLP (F) and IL-33 (G) in 6 week-old mouse whole lung homogenates were measured by ELISA one day after three daily intranasal injections of IL-33. (H) ILC2 numbers in postpubertal naïve *Il33*^-/-^ male and female mouse lungs. (I) Eosinophil and ILC2 numbers in IL-33 treated postpubertal WT and *Il33*^-/-^ lungs 3 days after 3 consecutive IL-33 administrations. Red = female, blue = male. Data represented are mean ± SEM, with 6–8 mice from 2–3 separate litters per group (A, C, D), 4 mice per group, 4 pictures per lung (B), 6–19 mice from 3–6 separate litters per group (E-G), 3 experiments with 3–8 mice per group (H) or 4 experiments with 7–17 mice per group (I). Two-tailed Student’s t-test was used to determine statistical significance, with a P value <0.05 being significant. *P<0.05, **P<0.01, ***P<0.005, ****P<0.0001.

Cephus et al. recently reported that IL-33 and TSLP are negatively regulated by male hormones in *Alternaria alternata* stimulated mice [15]. Because these cytokines are known to activate ILC2s, we measured the amounts of IL-33, TSLP and IL-7 in whole lung homogenate prepared from naïve male and female B6 mice at different ages. In young mice, the amount of IL-33 was similar in male and female lungs. Strikingly, the IL-33 levels increased at 6 weeks of age in female lungs, resulting in significantly higher amounts of endogenous IL-33 compared to male lungs (Fig 2A). Immunohistochemical analyses of naive lung sections also showed more IL-33 positive cells in 8 week-old female than male lungs (Fig 2B). The expression of IL-7 (Fig 2C) and TSLP (Fig 2D) also showed similar age dependent changes. IFNγ, a type 1 cytokine, was undetectable. We have also measured IL-7 and TSLP amounts after IL-33 injections to determine whether IL-33 induces differential expression of IL-7 and TSLP in male and female lungs. To this end, we selected 6 week-old mice as there is no significant difference in IL-7 and TSLP amounts between naïve male and female lungs at this age, and we can directly assess the effects of IL-33 on the amounts of IL-7 and TSLP. Intranasal injections of IL-33 into 6 week-old mice caused no significant change in IL-7 and TSLP amounts both in male and female mice (Fig 2E and 2F). The amount of IL-33 increased in male and female lungs after IL-33 administration, as expected, but the amount of IL-33 was no longer different between male and female (Fig 2G). To test whether the differences in the amount of endogenous IL-33 in naïve mice are responsible for the differences between female and male ILC2s in their responses to intranasal injections of IL-33, we compared female and male *Il33*^-/-^ mice. Similar to B6 mice, there was no difference in the numbers of ILC2s in post pubertal male and female *Il33*^*-/-*^ lungs before stimulation (Fig 2H). Three daily intranasal injections of IL-33 into post pubertal male and female *Il33*^-/-^ mice revealed statistically significant differences in the numbers and percentages of ILC2s and eosinophils between the sexes. Therefore, the differences in ILC2s between male and female are independent of endogenous IL-33 (Fig 2I, S2 Fig).

### Analyses of purified male and female lung ILC2s

**Fig 3:**
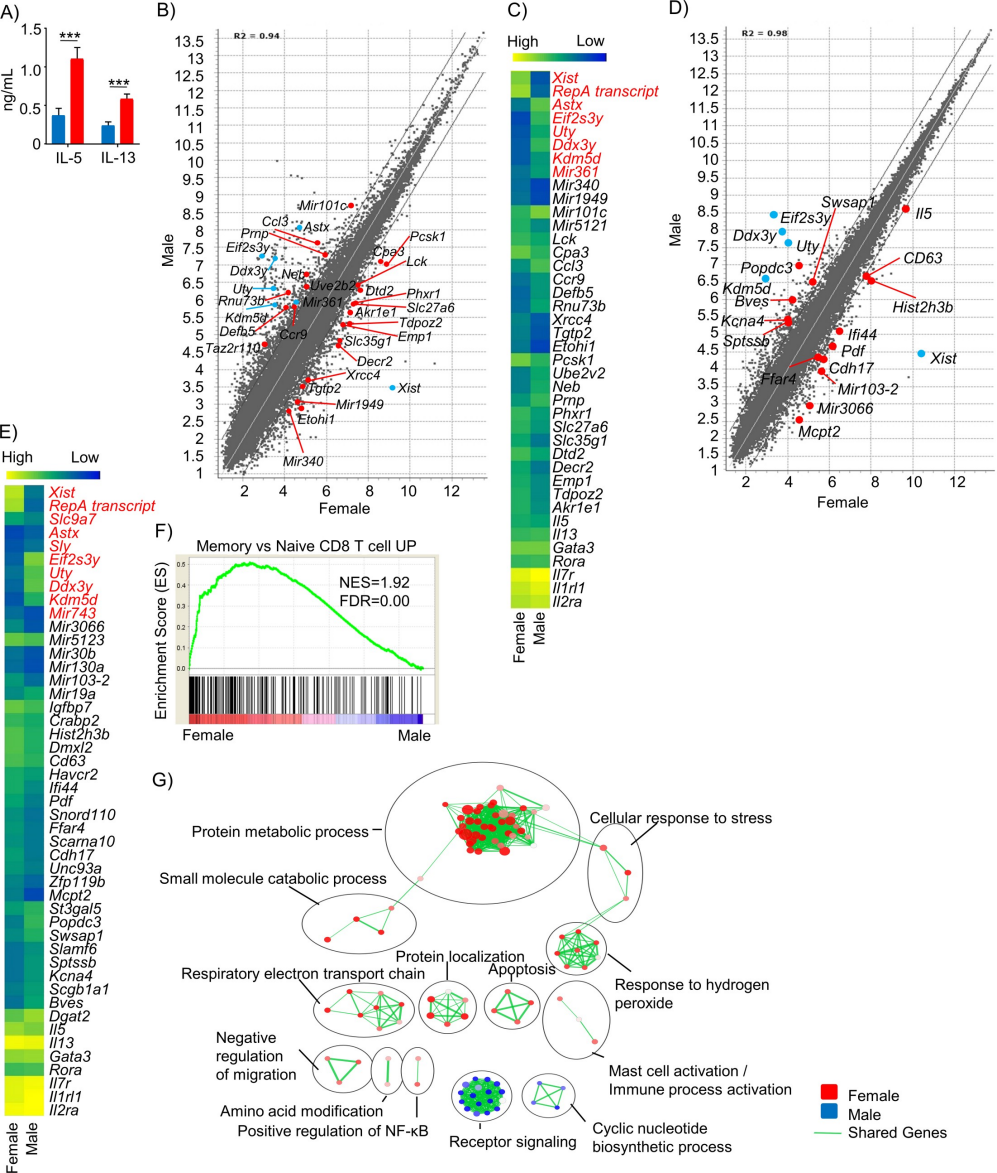
Female and male ILC2s have cell intrinsic differences. (A) Amounts of IL-5 and IL-13 produced by purified naïve 8 week old male or female ILC2s stimulated with 5 ng/ml IL-33 and TSLP after 48 h. Red = female, blue = male. Data represented are mean ± SEM, and representative of 2 independent experiments with 4–9 replicates per group. Two-tailed Student’s t-test was used to determine statistical significance, with a P value <0.05 being significant. ***P<0.005. (B) Scatter plot of 8-week-old naive male and female mouse lung ILC2 gene expressions in log2 scale. Black lines show fold change = 2. Blue = sex-linked genes, red = autosomal genes. Data represented are mean ± SEM, 5–14 mice per sample, 3 samples per group. (C) Relative expression levels of selected genes in 8-week-old naïve male and female mouse lung ILC2s. Annotated genes were selected if they were expressed at intermediate to high levels (>5.0 in log2 scale) in at least one sex and were more than 2 fold differentially expressed between male and female. Genes highlighted in red are sex-linked genes. (D) Scatter plot of gene expressions in male and female ILC2s purified from 8-week-old mouse lungs one day after three consecutive injections of IL-33. Gene expressions in log_2_ scale. Black lines show fold change = 2. Blue = sex-linked genes, red = autosomal genes. Data represented are mean ± SEM, 2 mice per sample, 3 samples per group. (E) Relative expression levels of selected genes in 8-week-old activated male and female mouse lung ILC2s. Genes highlighted in red are sex-linked genes. (F) GSEA of naïve male and female lung ILC2 microarray data in shown in (B). (G) BiNGO analyses comparing naïve female and male mouse lung ILC2 gene expressions. Cytoscape and EnrichmentMap were used for visualizing the functional clusters enriched in each sex and cellular state. Nodes represent enriched GO terms. Node size is proportional to total number of genes in gene set, and node colour to the (1-p value). Edges depict gene set overlap between nodes and thickness represents fraction of shared genes.

In vitro stimulation of ILC2s purified from 8 weeks old naïve male and female lungs demonstrated that female ILC2s produce more type 2 cytokines than male ILC2s (Fig 3A), demonstrating that female and male ILC2s differ in their ability to produce cytokines upon stimulation, in agreement with in vivo analyses (Fig 1F). The result also suggested a cell intrinsic difference between male and female ILC2s. To further investigate intrinsic sex differences in ILC2s, we performed microarray analyses of purified 8 weeks old naïve male and female lung ILC2s (S3A Fig). Overall, 4% of the genes were differentially expressed, the majority of which were autosomal rather than sex-linked genes (Fig 3B, S1 Table). Detailed analyses demonstrated no significant differences in the expression of *Il2ra* encoding CD25, *Il1rl1* encoding ST2, *Gata3*, *Rora*, *Il7r* encoding CD127, which are known to be important for ILC2 development or functions (Fig 3C). To test the functional relevance of some of the differentially expressed genes, we analyzed CCR9 expression on naïve male and female ILC2s by flow cytometry. Although majority of ILC2s did not expressed the receptor, there was significantly higher percentage of CCR9 expressing ILC2s in naïve male than female lung in accordance with gene expression analyses (S3B Fig). The mean fluorescence intensity (MFI) of CCR9 was also higher in naïve male than female ILC2s. We also analyzed chemokine CCL3 in BALF of naïve male and female. There was no significant difference in the amounts of CCL3 in BALF of male and female, which disagrees with gene expression data (S3C Fig). We also compared the gene expression profiles of ILC2s purified from IL-33 injected male and female (8 weeks old) lungs and found that they were remarkably similar to each other (Fig 3D). The number of differentially expressed genes in the activated ILC2s was much less than naïve female and male ILC2s (S3A Fig, S1 Table). Similar to naive male and female ILC2, there was no significant difference in the expression of *Il2ra*, *Il1rl1*, *Gata3*, *Rora*, and *Il7r*. However, in agreement with in vivo data, *Il5* was more highly expressed in activated female compared to male ILC2s (Fig 3E). Gene Set Enrichment Analysis (GSEA) of the naïve ILC2 microarray data set showed that female ILC2s are enriched for a gene signature of memory T cells, with leading edge genes including *Lck*, *Car5b*, *Manea*, *and Cmc1* (Fig 3F, S2 Table). To further understand the gene expression data, the set of genes that are differentially expressed between male and female ILC2s were analyzed for over-represented Gene Ontology (GO) terms and visualized as interaction networks using Cytoscape and Enrichment Map. Naive female ILC2s showed an over-representation of cellular metabolism-related networks, including protein metabolic process (S3 Table) and electron transport chain, implying that female ILC2s are more metabolically active than male ILC2s at naive state (Fig 3G). These results suggested that ILC2 intrinsic differences in gene expression may make naive female ILC2s more prone to be activated and proliferate than naive male ILC2s.

## Discussion

In this study, we have shown that postpubertal female ILC2s are more responsive to IL-33 stimulation than male counterpart using in vivo animal models. In vitro stimulation of purified ILC2s also demonstrated that female ILC2s have a greater ability to produce cytokines compared to male ILC2s upon stimulation with IL-33 and TSLP. Although transcriptome analysis of naive male and female lung ILC2s identified differentially expressed genes, we have not been able to find individual genes that are associated with ILC2 functions or development. On the other hand, GSEA of the same data set showed that the genes that are more highly expressed by naïve female than male ILC2s are enriched in a set of genes more highly expressed in memory than naive T cells. These results suggest that female ILC2s are more responsive to stimulation than male ILC2s, resembling the relationship between memory and naive T cells. Cytoscape network analyses of differentially expressed genes also suggest that female ILC2s may be more metabolically active than male ILC2s at naive state, consistent with the idea that female lung ILC2s are more prone to be activated, similar to memory T cells. Gene expression analyses of activated male and female ILC2s did not reveal specific genes that may play a role in the differences in male and female ILC2 responsiveness. Recent reports by Laffont et al., Cephus et al. and Kadel et al. showed that ILC2 development and their CD25 expression are negatively regulated by male sex hormones [14–16], explaining a potential mechanism behind epidemiologically observed sex-related differences in asthma prevalence. However, contradictory to those previous reports, we did not find a significant difference in lung ILC2 numbers (Fig 1C–1E) at any ages or strains tested, or the expression of *Il2ra* encoding CD25 (Fig 3C) between naïve male and female lung ILC2s. Differences in mouse strains, models and animal facilities, possibly including the microbial environment, may be responsible for the discrepancy in our results. It is also important to note that the number of ILC2s these studies detected in mouse lungs is noticeably higher than that that in our study, which suggest that tissue processing protocols or identification of ILC2s may also contribute to the discrepancy in our results.

We have also shown age-dependent differences in the expression of IL-33, IL-7 and TSLP between male and female lungs (Fig 2A, 2C and 2D). However, *Il33*^-/-^ mice showed similar sex-related differences in ILC2s (Fig 2I), indicating that the differences in the levels of IL-33 in naive male and female lungs were not responsible for the disparity in responsiveness between male and female ILC2s. Therefore, the significance of the differences in the amounts of IL-33, IL-7 and TSLP in naive male and female lungs in terms of ILC2 function is unclear. As activation of purified ILC2s by IL-33 in vitro requires an additional stimulus, which can be provided by IL-7 or TSLP [1], it seems likely that higher amounts of these cytokines in female than male mouse lungs may contribute to enhanced ILC2 activation in IL-33-injected female mice. It is interesting to note, however, that intranasal IL-33 injections did not cause upregulation of IL-7 (Fig 2E) or TSLP (Fig 2F). Furthermore, there is no significant difference in the amount of IL-33 in male and female lungs after IL-33 injections (Fig 2G). These results indicate that the higher levels of endogenous lung epithelial cytokines at steady state in females than males are unlikely to be responsible for the higher response of female ILC2s to IL-33 stimulation than male ILC2s. Our study suggested that multiple factors including ILC2 intrinsic and lung environmental components differ in male and female lungs. It is likely that sex differences in ILC2 responses and type 2 inflammation involve multiple mechanisms, and the effect of each component in isolation may not be significant.

Overall, our results have shown that postpubertal female ILC2s produce more cytokines upon stimulation than male ILC2s. It remains to be determined whether this is due to differences in gene expression potentially driven by the effects of sex hormones on ILC2s or the lung environment.

## Conclusions

Female ILC2s are more responsive than male ILC2s and naïve female mouse lungs have more ILC2 activating epithelial cytokines. These differences may contribute to the higher prevalence of non-allergic asthma in women than men. They also show the importance of considering sex and sex-related differences when studying asthma in animal models.

## Supporting information

S1 Fig Total cell counts and percentages of various cell types at different ages. (DOCX)

S2 Fig Total cell counts and percentages of various cell types in IL-33 KO and WT mice after IL-33 administration. (DOCX)

S3 Fig Gene expression analyses and validation by flow cytometry. (DOCX)

S1 Table Differentially expressed genes between male and female lung ILC2s. (XLS)

S2 Table Genes in the leading edge subset of the gene set shown in Fig 3F. (XLS)

S3 Table Genes that form the protein metabolic process cluster in the Cytoscape analyses. (XLSX)

## References

[1] Halim TY, Krauss RH, Sun AC, Takei F. Lung natural helper cells are a critical source of Th2 cell-type cytokines in protease allergen-induced airway inflammation. Immunity. 2012;36(3):451–63. Epub 2012/03/20. 10.1016/j.immuni.2011.12.020 . 22425247

[2] Bartemes KR, Iijima K, Kobayashi T, Kephart GM, McKenzie AN, Kita H. IL-33-responsive lineage- CD25+ CD44(hi) lymphoid cells mediate innate type 2 immunity and allergic inflammation in the lungs. Journal of immunology. 2012;188(3):1503–13. 10.4049/jimmunol.1102832 22198948 PMC3262877

[3] Martinez-Gonzalez I, Steer CA, Takei F. Lung ILC2s link innate and adaptive responses in allergic inflammation. Trends in immunology. 2015;36(3):189–95. 10.1016/j.it.2015.01.005 . 25704560

[4] Hamelmann E, Gelfand EW. IL-5-induced airway eosinophilia—the key to asthma? Immunological reviews. 2001;179:182–91. . 11292022 10.1034/j.1600-065x.2001.790118.x

[5] Cohn L. Mucus in chronic airway diseases: sorting out the sticky details. The Journal of clinical investigation. 2006;116(2):306–8. 10.1172/JCI27690 16453018 PMC1359062

[6] Allakhverdi Z, Comeau MR, Smith DE, Toy D, Endam LM, Desrosiers M, et al CD34+ hemopoietic progenitor cells are potent effectors of allergic inflammation. The Journal of allergy and clinical immunology. 2009;123(2):472–8. 10.1016/j.jaci.2008.10.022 . 19064280

[7] Bartemes KR, Kephart GM, Fox SJ, Kita H. Enhanced innate type 2 immune response in peripheral blood from patients with asthma. The Journal of allergy and clinical immunology. 2014;134(3):671–8 e4. 10.1016/j.jaci.2014.06.024 25171868 PMC4149890

[8] Christianson CA, Goplen NP, Zafar I, Irvin C, Good JT Jr., Rollins DR, et al Persistence of asthma requires multiple feedback circuits involving type 2 innate lymphoid cells and IL-33. The Journal of allergy and clinical immunology. 2015;136(1):59–68 e14. 10.1016/j.jaci.2014.11.037 25617223 PMC4494983

[9] Leynaert B, Sunyer J, Garcia-Esteban R, Svanes C, Jarvis D, Cerveri I, et al Gender differences in prevalence, diagnosis and incidence of allergic and non-allergic asthma: a population-based cohort. Thorax. 2012;67(7):625–31. 10.1136/thoraxjnl-2011-201249 . 22334535

[10] Melgert BN, Postma DS, Kuipers I, Geerlings M, Luinge MA, van der Strate BW, et al Female mice are more susceptible to the development of allergic airway inflammation than male mice. Clinical and experimental allergy: journal of the British Society for Allergy and Clinical Immunology. 2005;35(11):1496–503. 10.1111/j.1365-2222.2005.02362.x . 16297148

[11] Blacquiere MJ, Hylkema MN, Postma DS, Geerlings M, Timens W, Melgert BN. Airway inflammation and remodeling in two mouse models of asthma: comparison of males and females. International archives of allergy and immunology. 2010;153(2):173–81. 10.1159/000312635 . 20413985

[12] Takeda M, Tanabe M, Ito W, Ueki S, Konnno Y, Chihara M, et al Gender difference in allergic airway remodelling and immunoglobulin production in mouse model of asthma. Respirology. 2013;18(5):797–806. 10.1111/resp.12078 . 23490273

[13] Warren KJ, Sweeter JM, Pavlik JA, Nelson AJ, Devasure JM, Dickinson JD, et al Sex differences in activation of lung-related type 2 innate lymphoid cells in experimental asthma. Annals of allergy, asthma & immunology: official publication of the American College of Allergy, Asthma, & Immunology. 2017;118(2):233–4. 10.1016/j.anai.2016.11.011 28017508 PMC5291757

[14] Laffont S, Blanquart E, Savignac M, Cenac C, Laverny G, Metzger D, et al Androgen signaling negatively controls group 2 innate lymphoid cells. The Journal of experimental medicine. 2017;214(6):1581–92. 10.1084/jem.20161807 28484078 PMC5461006

[15] Cephus JY, Stier MT, Fuseini H, Yung JA, Toki S, Bloodworth MH, et al Testosterone Attenuates Group 2 Innate Lymphoid Cell-Mediated Airway Inflammation. Cell reports. 2017;21(9):2487–99. 10.1016/j.celrep.2017.10.110 29186686 PMC5731254

[16] Kadel S, Ainsua-Enrich E, Hatipoglu I, Turner S, Singh S, Khan S, et al A Major Population of Functional KLRG1(-) ILC2s in Female Lungs Contributes to a Sex Bias in ILC2 Numbers. ImmunoHorizons. 2018;2(2):74–86. 10.4049/immunohorizons.1800008 29568816 PMC5860819

[17] Bartemes K, Chen CC, Iijima K, Drake L, Kita H. IL-33-Responsive Group 2 Innate Lymphoid Cells Are Regulated by Female Sex Hormones in the Uterus. Journal of immunology. 2018;200(1):229–36. 10.4049/jimmunol.1602085 29133293 PMC5736420

[18] Halim TY, Takei F. Isolation and characterization of mouse innate lymphoid cells. Current protocols in immunology / edited by Coligan John E [et al]. 2014;106:3 25 1–13. 10.1002/0471142735.im0325s106 . 25081911

[19] Steer CA, Martinez-Gonzalez I, Ghaedi M, Allinger P, Matha L, Takei F. Group 2 innate lymphoid cell activation in the neonatal lung drives type 2 immunity and allergen sensitization. The Journal of allergy and clinical immunology. 2017;140(2):593–5 e3. 10.1016/j.jaci.2016.12.984 . 28216436

[20] Maere S, Heymans K, Kuiper M. BiNGO: a Cytoscape plugin to assess overrepresentation of gene ontology categories in biological networks. Bioinformatics. 2005;21(16):3448–9. 10.1093/bioinformatics/bti551 . 15972284

[21] Merico D, Isserlin R, Stueker O, Emili A, Bader GD. Enrichment map: a network-based method for gene-set enrichment visualization and interpretation. PloS one. 2010;5(11):e13984 10.1371/journal.pone.0013984 21085593 PMC2981572

[22] Rigas D, Lewis G, Aron JL, Wang B, Banie H, Sankaranarayanan I, et al Type 2 innate lymphoid cell suppression by regulatory T cells attenuates airway hyperreactivity and requires inducible T-cell costimulator-inducible T-cell costimulator ligand interaction. The Journal of allergy and clinical immunology. 2017;139(5):1468–77 e2. 10.1016/j.jaci.2016.08.034 27717665 PMC5378695

[23] Molofsky AB, Van Gool F, Liang HE, Van Dyken SJ, Nussbaum JC, Lee J, et al Interleukin-33 and Interferon-gamma Counter-Regulate Group 2 Innate Lymphoid Cell Activation during Immune Perturbation. Immunity. 2015;43(1):161–74. 10.1016/j.immuni.2015.05.019 26092469 PMC4512852

[24] Ahnstedt H, Roy-O'Reilly M, Spychala MS, Mobley AS, Bravo-Alegria J, Chauhan A, et al Sex Differences in Adipose Tissue CD8(+) T Cells and Regulatory T Cells in Middle-Aged Mice. Frontiers in immunology. 2018;9:659 10.3389/fimmu.2018.00659 29670627 PMC5893719

[25] Afshan G, Afzal N, Qureshi S. CD4+CD25(hi) regulatory T cells in healthy males and females mediate gender difference in the prevalence of autoimmune diseases. Clin Lab. 2012;58(5–6):567–71. . 22783590

