# Correction: Female and male mouse lung group 2 innate lymphoid cells differ in gene expression profiles and cytokine production

**DOI:** 10.1371/journal.pone.0252257

**Published:** 2021-05-20

**Authors:** Laura Mathä, Hanjoo Shim, Catherine A. Steer, Yi Han Yin, Itziar Martinez-Gonzalez, Fumio Takei

In the Antibodies, reagents and flow cytometers subsection of the Materials and methods, there is an error in the first sentence of the first paragraph. The correct sentence is: Fluorescein isothiocyanate (FITC)-conjugated anti-Ki67 (SolA15), Rat IgG2a kappa isotype control (eBR2a), PerCP-Cy5.5-conjugated anti-CD19 (1D3), NK1.1 (PK136), CD3ε (145-2C11), CD25 (PC61.5), Allophycocyanin (APC)-conjugated anti-FcεR1α (MAR-1), CCR9 (CW-1.2), Mouse IgG2a kappa isotype control (eBM2a), Alexa Fluor 700-conjugated anti-CD45.2 (104), CD11c (N418), eFluor 450-conjugated anti-CD3ε (145-2C11), CD4 (RM4-5), CD19 (1D3), CD11b (M1/70), TCRγδ (GL3), CD11c (N418), NK1.1 (PK136), TCRβ (H57-597), Gr1 (RB6-8C5), Ter119 (TER-119), Phycoerythrin (PE)-conjugated anti-CD127 (A7R34) and anti-GATA3 (TWAJ), PE-Cyanine7 conjugated anti-CD127 (A7R34) were purchased from Thermo Fisher Scientific (Waltham, MA).

In the In vitro stimulation subsection of the Materials and methods, there is an error in the first sentence of the first paragraph. The correct sentence is: ILC2s were sorted from naïve female and male lungs after ILC2 enrichment and 1000 cells were cultured in 200 μL RPMI-1640 media containing 10% FBS, P/S, 50 μM 2ME, 5 ng/mL TSLP and IL-33.

In Fig 1, the Y axis labels in images C, G, I, and N are not properly aligned. The authors have provided a corrected version here.

**Fig 1:**
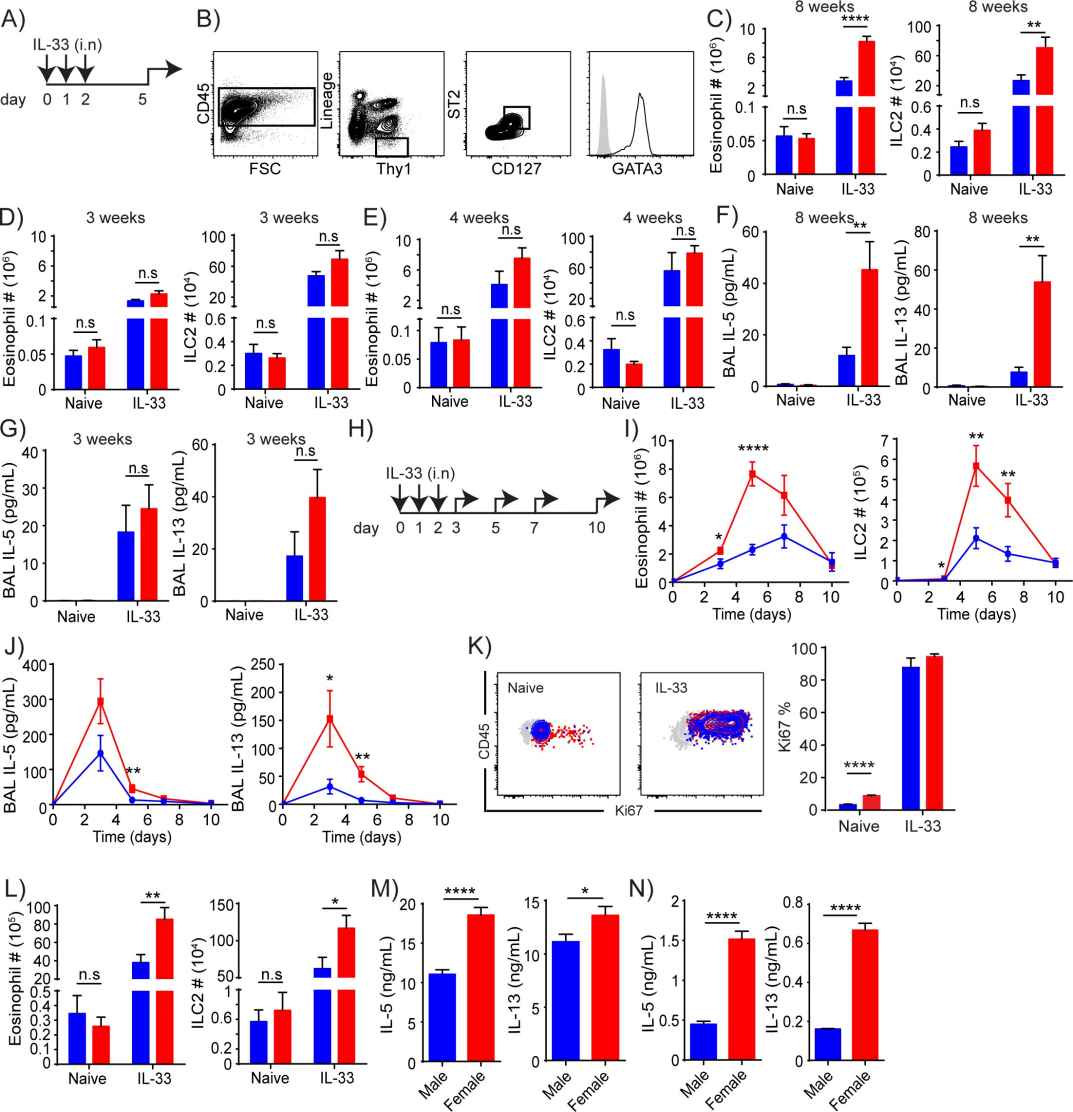
Female ILC2s respond more vigorously to IL-33 stimulation than male ILC2s. (A) Treatment scheme for in vivo experiments. (B) Gating strategy to identify ILC2s. They were identified as Lineage (CD3ε, CD4, CD19, CD11b, CD11c, TCRβ, TCRγδ, NK1.1, Gr1, Ter119)^-^CD45^+^Thy1(CD90)^+^CD127^+^ST2^+^ cells and confirmed for GATA3 expression. Grey = fluorescence-minus-one control, black line = ILC2. (C-E) Eosinophil and ILC2 numbers in untreated or IL-33 treated 8 week- (C), 3 week- (D) and 4 week- (E) old mice 3 days after 3 consecutive IL-33 injections. (F,G) BALF cytokines in untreated or IL-33 treated 8 week- (F) and 3 week- (G) old mice 3 days after 3 consecutive IL-33 injections. (H) Treatment scheme for in vivo time-course analyses. (I, J) Eosinophil and ILC2 numbers (I) and BALF cytokines (J) were quantified at day 0 (naive) and then 1 day (day 3), 3 days (day 5), 5 days (day 7) and 8 days (day 10) after 3 consecutive IL-33 injections. (K) Ki67 staining of male (blue) and female (red) ILC2s before and 1 day after IL-33 injections. Grey = Isotype control. (L) Eosinophil and ILC2 numbers in untreated or IL-33 treated 8 week-old *Rag1*^*-/-*^ mice 3 days after 3 consecutive IL-33 administrations. (M, N) Amounts of IL-5 and IL-13 in supernatant collected from male or female B6 (M) or *Rag1*^-/-^ (N) whole lung leukocytes cultures stimulated with 10 ng/ml (M) or 5 ng/ml (N) IL-33 and TSLP for 72 hours. Red = female, blue = male. Data represented are mean ± SEM of more than 3 (C, D, F, G, I, J) or 2 (E, K, L) experiments with 5–14 (C, D, F, G), 4–19 (I, J), 4–6 (E), 5–7 (K) or 3–7 (L) mice per group, or mean ± SEM of 7–10 (M) or 4–5 (N) replicates per group. Two-tailed Student’s t-test was used to determine statistical significance, with a P value <0.05 being significant. *P<0.05, **P<0.01, ****P<0.0001, ns, not significantly different [P>0.05].
