# Supplementary material for: Female and male mouse lung group 2 innate lymphoid cells differ in gene expression profiles and cytokine production
Source: PLoS One. 2019 Mar 26;14(3):e0214286. doi: 10.1371/journal.pone.0214286 (PMC6435236; doi:10.1371/journal.pone.0214286)
Supplement: S1 Fig — (DOCX) [file pone.0214286.s001.docx]

**
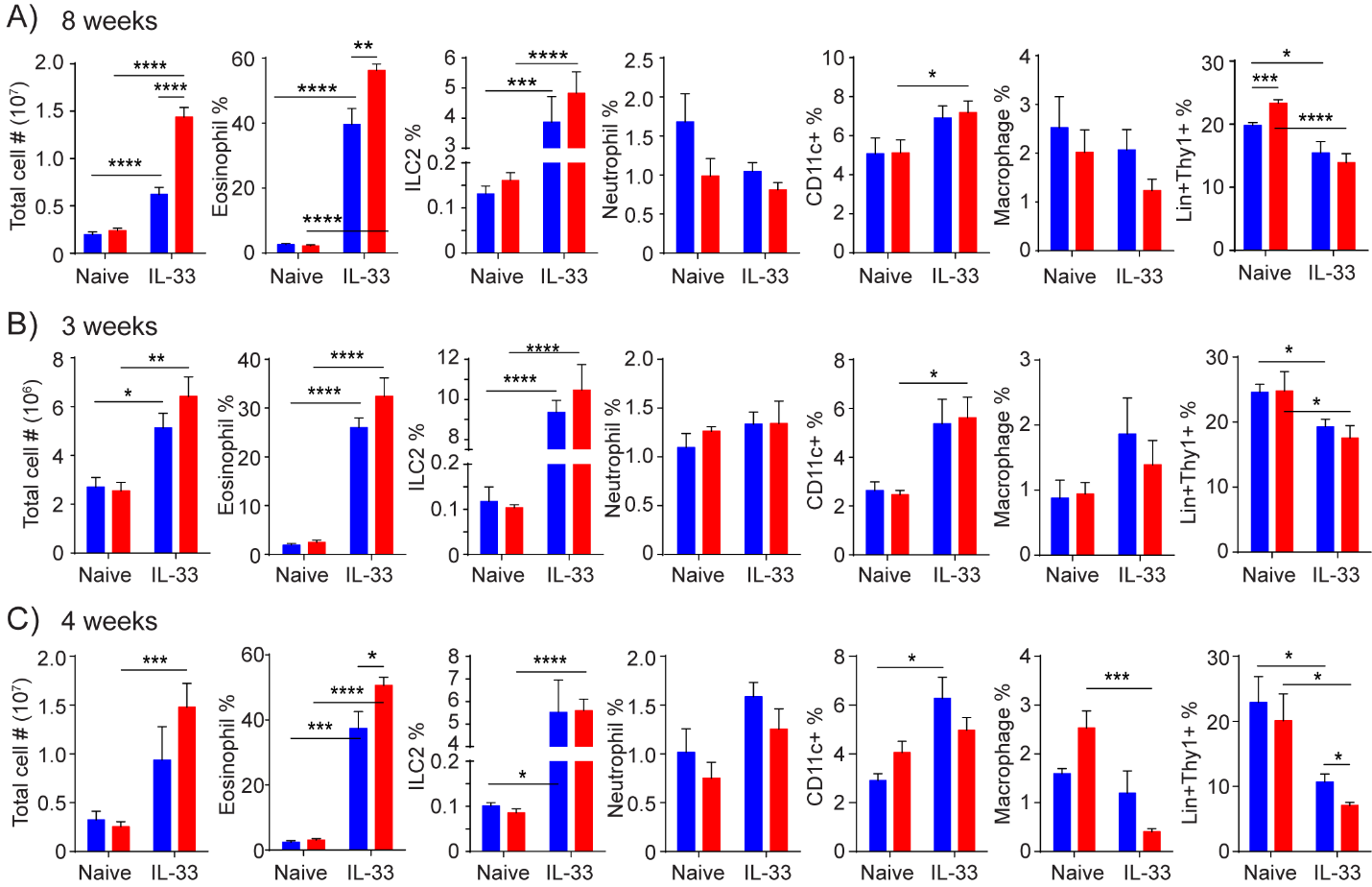
S1 Fig. Total cell counts and percentages of various cell types at different ages.**

(A-C) Naïve or IL-33 treated mice were analyzed on day 5 (treatment scheme in Fig 1 A). Total cell counts and percentages (out of CD45^+^ cells) of different immune populations in male (blue) and female (red) lungs at 8 weeks (A), 3 weeks (B) and 4 weeks (C) of age. Eosinophils and ILC2s were identified as indicated in materials and methods. Neutrophils and macrophages were identified as shown in reference [[1](#_ENREF_1)]. CD11c^+^ cells were identified as CD45^+^CD11c^+^ cells and Lin^+^Thy1^+^ population was identified as CD45^+^Lin^+^Thy1^+^ cells. Data represented are mean ± SEM of more than 3 (C, D) or 2 (E) experiments with 5-14 (C, D) or 4-6 (E) mice per group. Two-tailed Student’s t-test was used to determine statistical significance, with a P value <0.05 being significant. *P<0.05, **P<0.01, **P<0.001, ****P<0.0001.

**Reference:**

1. Halim TY, MacLaren A, Romanish MT, Gold MJ, McNagny KM, et al. (2012) Retinoic-acid-receptor-related orphan nuclear receptor alpha is required for natural helper cell development and allergic inflammation. Immunity 37: 463-474.
