# Supplementary material for: Female and male mouse lung group 2 innate lymphoid cells differ in gene expression profiles and cytokine production
Source: PLoS One. 2019 Mar 26;14(3):e0214286. doi: 10.1371/journal.pone.0214286 (PMC6435236; doi:10.1371/journal.pone.0214286)
Supplement: S2 Fig — (DOCX) [file pone.0214286.s002.docx]

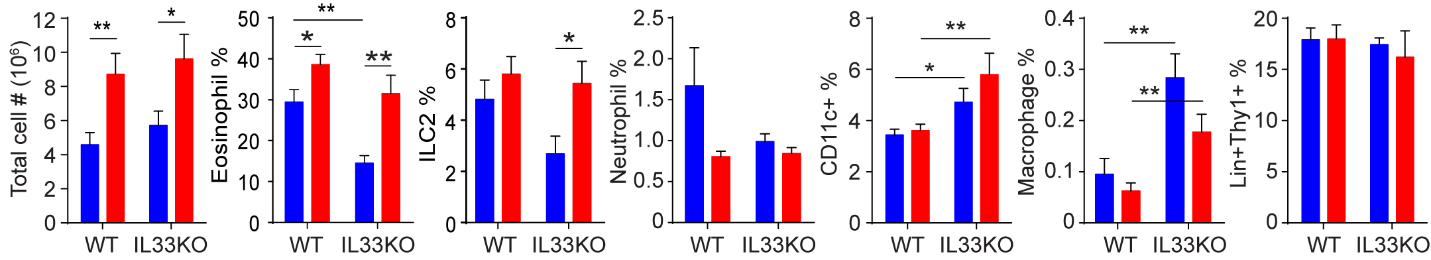


**S2 Fig. Total cell counts and percentages of various cell types in IL-33 KO and WT mice after IL-33 administration.**

IL-33 treated WT or IL-33 KO mice were analyzed on day 5 after treatment (treatment scheme in Fig 1 A). Eosinophils and ILC2s were identified as indicated in materials and methods. Neutrophils and macrophages were identified as shown in reference [[1](#_ENREF_1)]. CD11c^+^ cells were identified as CD45^+^CD11c^+^ cells and Lin^+^Thy1^+^ population was identified as CD45^+^Lin^+^Thy1^+^ cells. Data represented are mean ± SEM, 4 experiments with 7-17 mice per group. Two-tailed Student’s t-test was used to determine statistical significance, with a P value <0.05 being significant. *P<0.05, **P<0.01.

**Reference:**

1. Halim TY, MacLaren A, Romanish MT, Gold MJ, McNagny KM, et al. (2012) Retinoic-acid-receptor-related orphan nuclear receptor alpha is required for natural helper cell development and allergic inflammation. Immunity 37: 463-474.
