# Supplementary material for: Female and male mouse lung group 2 innate lymphoid cells differ in gene expression profiles and cytokine production
Source: PLoS One. 2019 Mar 26;14(3):e0214286. doi: 10.1371/journal.pone.0214286 (PMC6435236; doi:10.1371/journal.pone.0214286)
Supplement: S3 Fig — (DOCX) [file pone.0214286.s003.docx]

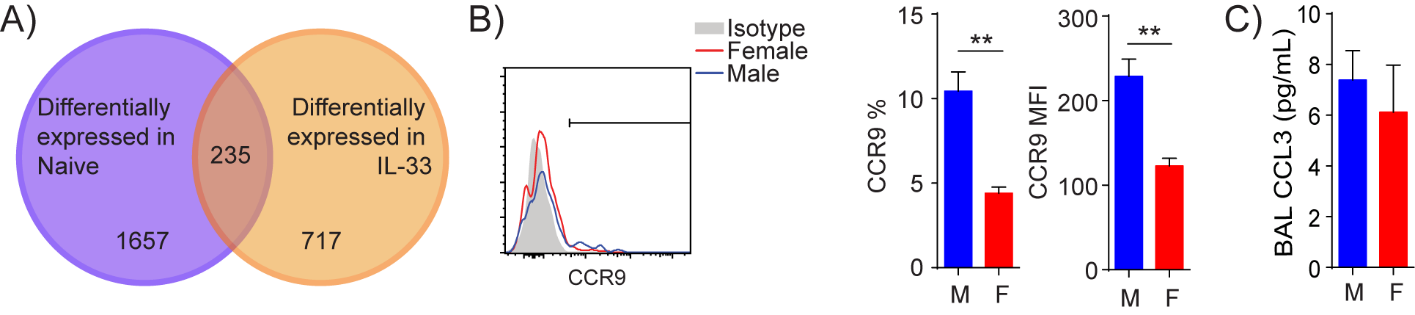


**S3 Fig. Gene expression analyses and validation by flow cytometry.**

(A) a Venn diagram summarizing the numbers of differentially expressed genes between naïve and IL-33 treated male and female ILC2s one day after three daily injections. (B) Expression of CCR9 by male and female ILC2s in histogram (left, y axis in modal scale), % of CCR9 positive cells (middle) and mean fluorescence intensity (MFI) (right). Blue=male, Red=female. (C) The amounts of CCL3 in naïve mouse BALF. Blue=male, Red=female. Data represented are mean ± SEM, 5-14 mice per sample, 3 samples per group (A), 2 experiments with 5-6 mice per group (B) or 3 experiments with 10-11 mice per group (C). Two-tailed Student’s t-test was used to determine statistical significance, with a P value <0.05 being significant. **P<0.01.
